# Evaluating the Treatment Efficacy of Nano-Drug in a Lung Cancer Model Using Advanced Functional Magnetic Resonance Imaging

**DOI:** 10.3389/fonc.2020.563932

**Published:** 2020-09-29

**Authors:** Cuiqing Huang, Jianye Liang, Mengjie Ma, Qingqing Cheng, Xi Xu, Dong Zhang, Changzheng Shi, Ning Shang, Zeyu Xiao, Liangping Luo

**Affiliations:** ^1^Medical Imaging Center, The First Affiliated Hospital of Jinan University, Guangzhou, China; ^2^Ultrasound Department, Guangdong Province Women and Children’s Hospital, Guangzhou, China; ^3^Department of Medical Imaging, Sun Yat-sen University Cancer Center, State Key Laboratory of Oncology in South China, Collaborative Innovation Center for Cancer Medicine, Guangzhou, China

**Keywords:** tumor microenvironment, nanomedicine, lung cancer, R2^∗^ mapping, intravoxel incoherent motion, diffusion-weighed imaging

## Abstract

**Objectives:**

Nano-drug delivery system is an interesting field in precise cancer treatment, but few study has reported the microenvironmental changes after such treatment. This study aimed to detect the hemodynamic and microenvironmental changes in a lung cancer xenograft model after treated with doxorubicin (DOX) encapsulated by a cyclic arginine-glycine-aspartic acid polypeptide modified poly-(lactic-co-glycolic acid) nanosystem (cRGD-PLGA@DOX) using functional magnetic resonance imaging.

**Materials and Methods:**

Thirty-two tumor-bearing mice were randomly divided into four groups. Group A was treated with 0.9% saline, Group B with 4 mg/kg of doxorubicin, Group C with 2 mg/kg of cRGD-PLGA@DOX, and Group D with 4 mg/kg of cRGD-PLGA@DOX. Intravoxel incoherent motion diffusion-weighed imaging (IVIM-DWI) and R2^∗^ mapping were performed, and D^∗^, f, D, and R2^∗^ values were obtained before and1, 2, and 3 weeks after treatment. They were sacrificed for pathological examination after examinations.

**Results:**

The reconstructed cRGD-PLGA@DOX was homogeneous, well-dispersed, and spherical in shape, with an average size of 180 nm. Group D demonstrated the smallest tumor volume and highest tumor inhibition rate in 3 weeks. D value of Group B, C, and D manifested an upward trend in 3 weeks with the highest increase in Group D. D^∗^ values shared a similar increased trends with f values in Group A, B, and C in 3 weeks, except Group D. R2^∗^ value of Group A gradually increased in 3 weeks, but the trends were reversed in the treatment groups. D value was significantly negative with Ki-67 expression (*r* = −0.757, *P* < 0.001) but positive with TUNEL (*r* = 0.621, *P* < 0.001), and phosphate and tension homology deleted on chromosome ten (PTEN) staining (*r* = 0.57, *P* = 0.004). R2^∗^ value was closely correlated with HIF-1a (*r* = 0.721, *P* < 0.001).

**Conclusion:**

The nano-drug demonstrated an enhanced anti-tumor effect without the need of increased chemotherapeutic dosage. The tumor microenvironment such as cellular and perfusion changes during treatment can be non-invasively detected by two functional MRI including IVIM-DWI and R2^∗^ mapping.

## Introduction

Lung cancer is the most commonly diagnosed cancer (11.6% of the total cases) and the leading cause of cancer death (18.4% of the total cancer deaths) in 2018 around the world (1). The incidence and mortality of lung cancer still increased in recent 30 years. A combined chemotherapy is the main treatment modality of lung cancer in addition to surgery. However, a variety of side-effects may arise due to the low targeted characteristics and high tissue toxicity, reducing the tolerance of receiving further therapy. Therefore, more specific agents are needed to reduce the side effect and improve therapeutic efficacy (2). In recent years, the rapid development of nanotechnology has provided new opportunities for cancer treatment with nanomedicine. The drug delivery system is designed based on the difference between the microenvironment and tissue structure between normal and tumor tissues. The endothelial gaps in normal tissues are dense and the vascular walls are structurally intact, preventing the macromolecules or lipid particles from penetrating into the interstitial space. In solid tumor tissue, there are abundant immature blood vessels with incontinuous endothelial cells, as well as blocked lymphatic reflux, causing an enhanced permeability and retention effect for nanoparticles, which are the most commonly used mechanisms for passively targeting the tumors in nanomedicine (3). The nanoparticles also have other superiorities such as good biocompatibility, biodegradability, surface programmability for active targeting, and a superior payload release kinetics profile (4, 5). How to effectively monitor the tumor microenvironment is an important key to reflect the therapeutic efficacy of nanomedicine during anti-tumor treatment.

In recent decades, magnetic resonance imaging (MRI) has become a valuable imaging technique for the cancer diagnosis and treatment evaluation with high soft tissue resolution, non-invasive and multiparametric characteristics. Intravoxel incoherent motion diffusion-weighted imaging (IVIM-DWI) which was first introduced by Le Bihan et al. (6), can separate the incoherent motion of water molecules within the capillaries from extravascular molecular diffusion. The true diffusion coefficient (D value), pseudo-diffusion coefficient (D^∗^ value), and perfusion fraction (f value) were generated using a bi-exponential model with multiple b-value (7). It can provide the functional information regarding the tumor cellularity and microcirculation perfusion without the need of contrast agent, and therefore can be repetitively performed within a short time interval (8). R2^∗^ mapping was another non-invasive sequence that is sensitive to paramagnetic substances such as deoxyhemoglobin, reflecting the degree of tumor hypoxia (9, 10). Previous studies had confirmed the values of these sequences in monitoring the tumor microenvironment during anti-vascular treatment in multiple animal models (10–12), but few studies have established the relationships between these functional sequences and nanoparticle targeted therapy, which should be much helpful for nanomedicine development.

Doxorubicin (DOX) has been approved by the FDA as an effective antitumor agent with a wide antitumor spectrum. It also induces a various degree of cardiac, renal, and medullary toxicities and arouses drug resistance in the standard chemotherapy. The poly-(lactic-co-glycolic acid) (PLGA) polymer micelles have been regarded as an ideal functional nanocarrier, providing an active targeting effect for the drugs to the tumor cells after conjugated with specific ligand or antibody (13, 14). Cyclic arginine-glycine-aspartic acid polypeptide (cRGD) is such a tripeptide ligand that specifically binds to the integrin αvβ3, a receptor which is highly expressed on various kinds of tumors including lung cancer (15, 16). In this study, a cRGD modified PLGA copolymer was constructed to encapsulate doxorubicin (cRGD-PLGA@DOX) to improve local drug delivery and reduce systemic toxicities. We compared the therapeutic efficacy of this nano-drug with the unencapsulated doxorubicin and also in two different dosages. The microenvironment changes regarding tumor cellularity, hypoxia and microcirculation perfusion during treatments were monitored by two functional sequences including IVIM-DWI and R2^∗^ mapping. The results should help us more comprehensively illuminate the values of nanomedicine in cancer treatment in a molecular level.

## Materials and Methods

### Synthesis and Characterization of Nano-Drug

The cRGD-PLGA@DOX nanoparticle was constructed by the College of Chemistry and Materials Science of Jinan University, following methods published in a previous study (3). Briefly, cRGD was conjugated with polyethylene glycol (PEG)–polyetherimide–lactic acid–hydroxyacetic acid to establish the basic frame of the copolymer, then doxorubicin was dissolved into the multipolymer using emulsification and solvent evaporation techniques. Doxorubicin was obtained from the first affiliated hospital of Jinan University. The transmission electron microscopy (TEM) images of the nanoparticles were observed using the JEOL Model JEM-2010 system at an accelerating voltage of 200 kV. The nanoparticles were dropped onto porous carbon films on copper grid, followed by air drying prior to imaging. We measured the size distribution and zeta potential of the nanoparticles with a Zetasizer Nano ZS 90 particle analyzer (Malvern Instruments Ltd., Worcestershire, United Kingdom). The dispersion of nanoparticles was diluted with ultrapure water and subsequently analyzed. The doxorubicin concentration in nanoparticle was measured by RP-HPLC on a JASCO HPLC system equipped with a Vydac C18 analytical (5 μm) column. Isocratic chromatography conditions included a mobile phase of acetonitrile and water (55:45 v/v) with a flow rate of 1.0 mL/min. The nanoparticle was dispersed in phosphate buffer solution (PBS, pH 5.3, 6.0, and 7.4), lysozyme solution or cell lysate solution with constant shaking in dark tubes at 37 °C. A 300 μL aliquot was collected and replaced with fresh solution at a certain interval. The stability of cRGD-PLGA@DOX nanoparticle was measured under PBS (pH 7.4) and human serum within 72 h.

### Cell Culture

The A549 cell lines of human non-small cell lung cancer were obtained from the American Type Culture Collection (ATCC, Manassas, VA) and cultured in a 90% RPMI-1640 medium supplemented with 10∼20% FBS and 1% penicillin/streptomycin, and maintained at 37°C in a 5% CO_2_ atmosphere.

### MTT Assays

The MTT assay was applied to determine the cytotoxicity of cRGD-PLGA@DOX. A549 and HeLa cells were seeded at 2 × 10^3^ cells/well into a 96-well plate and maintained for 24 h. Free DOX and cRGD-PLGA@DOX at DOX doses of 0.125–8 μM were added to the wells. After incubation for 72 h, 20 μL/well of MTT solution was added and incubated for 6 h at 37°C. And the microplate spectrophotometer (SpectroA maxTM250) was utilized to measure the cytotoxicity (the absorbance at 570 nm).

### Tumor Model and Grouping

The Institutional Animal Ethics and Use Committee of Jinan University approved the animal experiments. We strictly followed the Institutional Laboratory Animal Care and Use Manual. A total of 40 male BALB/c nude mice (aged 6–7 weeks and weighed about 20 g), were purchased from Vital River Laboratory Animal Technology Corporation (Beijing, China) and raised in a specific pathogen-free environment. The mice were subcutaneously injected with 1 × 10^6^/ml A549 cells in 0.2 ml of serum-free media mixed with high concentration matrigel into the right flank near the hindlimbs to develop the lung cancer model. Experiments were initiated after 15 days when the tumor reached a transverse diameter of approximately 8–10 mm as the tumors maintained a rapid growth stage with relatively high vascularization and without obvious necrosis, tending to be the best opportunity to observe the treatment effect arising from the drugs. Thirty-two tumor-bearing mice were selected and randomly divided four groups for eight in each group. They were treated with normal saline (Group A), 4 mg/kg of doxorubicin (Group B), 2 mg/kg (Group C), and 4 mg/kg of cRGD-PLGA@DOX (Group D) via the tail vein once every 2 days. The tumor volume was calculated using the following formula: (a^2^ × b × 0.5) mm^3^, where a refers to the smaller diameter and b is the diameter perpendicular to a, measured with a slide caliper (17). The tumor inhibition rate refers to (tumor size of untreated mice−tumor size of treated mice)/tumor size of untreated mice × 100%.

### MRI Examinations

The mice were anesthetized through intraperitoneal injection with 0.2% pentobarbital sodium before scanning. They were imaged in the supine position before and in 1, 2, and 3 weeks after treatment using a 1.5 T Signa HDxt superconductor clinical MR system (GE Medical System, Milwaukee, WI) equipped with a four-channel animal coil. Fast spin-echo sequence was used to perform T1-weighted imaging (T1WI) with following parameters: repetition time/echo time (TR/TE) of 540/14.7 ms, slice thickness of 2 mm, slice gap of 0.2 mm, field of view (FOV) of 5 cm× 5 cm, matrix size of 192 × 160, number of excitations (NEX) of 2. The parameters of T2WI were as follows: TR/TE of 2280/77.6 ms, slice thickness of 2 mm, slice gap of 0.2 mm, FOV of 5 cm × 5 cm, matrix size of 192 × 160 and NEX of 2. The IVIM-DW MR images were obtained using a single-shot, echo-planar imaging pulse sequence with chemical shift-selective saturation technique for fat suppression. Three orthogonal directions were set as the diffusion gradients including thirteen b-values: 0, 25, 50, 75, 100, 150, 200, 400, 600, 800, 1000, 1200, 1500 s/mm^2^), and TR/TE of 4200/101.7 ms, matrix size of 96 × 128 and FOV of 7.0 × 5.6 cm^2^. The parameters of R2^∗^ mapping were as follows: sixteen TEs (TE = 3.4, 9.3, 15.2, 21.2, 27.1, 33, 38.9, 44.8, 50.7, 56.6, 62.5, 68.5, 74.4, 80.3, 86.2, 92.1), TR of 160 ms, FOV of 8.0 cm × 6.4 cm, matrix size of 192 × 128 and NEX of 2.

### Image Post-processing

All IVIM-DWI data were transferred to a dedicated post-processing workstation (AW4.5, GE Health Care, United States) using the Functool-MADC software. The equation of the bi-exponential model was expressed as SI/SI_0_ = (1−f) ⋅ exp(−bD) + f ⋅ exp(−bD^∗^). SI_0_ refers to the mean signal intensity of the region of interest (ROI) for a b-value of 0 s/mm^2^ while SI refers to the signal intensity for higher b-values. b-value means the diffusion sensitivity coefficient. D values represent the pure diffusion of water molecule, also known as true diffusion coefficient, D^∗^ values refer to pseudo-diffusion, which calculate the amount of microcirculation perfusion. F value is the perfusion fraction, standing for the percentage of microcirculation perfusion among the signal decay from total diffusion effect. A segmented method was used to fit the bi-exponential IVIM model. First, as b-value less than 200 mm^2^/s was referred to low b-value where mainly reflected the pseudo-diffusion, and the data in this ranges were fitted to the bi-exponential model for acquiring D^∗^ and f values. Then the data of b-values higher than 200 mm^2^/s were used to obtain D value using a mono-exponential model, because the pseudo-diffusions from blood flows were negligible in this region (18). The Functool-R2Star software was applied to process the BOLD-MRI data for generating the transverse relaxation rate (R2^∗^). Theoretically, R2^∗^ maps were generated by linearly fitting a single exponential model of the ln(signal intensity) to TE curve. The slope of ln(signal intensity) vs TE determines the R2^∗^ (l/T2^∗^) values (19). T2WI was referenced to delineate the tumor areas at the largest cross section as the ROI but avoiding encompassing the skins and muscles.

### Histological Analysis

After the last examinations, all the mice were sacrificed and their tumor samples were removed for pathologic examinations. The tumor sample was fixed in 4% paraformaldehyde for 24 h. They were embedded in paraffin and sectioned at 5 μm thickness for subsequent staining. Haematoxylin and eosin (HE) staining, hypoxia-inducible factor 1α (HIF-1α), Ki-67, TUNEL, and PTEN immunofluorescent staining were performed to analyze the pathological changes. The antibodies were all purchased from Servicebio Technology CO., LTD. (Wuhan, China). The expression of HIF is activated by hypoxia condition, which can be detected by monoclonal anti-HIF-1α antibodies, and is generally used to assess the tumor hypoxia. The procedures of HIF-1α staining were referenced to the study of Li et al. (20). Ki-67 is a proliferation marker reflecting the degree of proliferation activity of tumor cells. The cell apoptosis was evaluated by terminal-deoxynucleoitidyl transferase mediated nick end labeling (TUNEL) staining using an *in situ* Cell Death Detection Kit following the manufacturer’s instruction. PTEN is an important tumor suppressor gene that inhibits the tumor growth by antagonizing the activity of phosphorylase. An Olympus BX 53 microscope was used to observe the staining slices. Three high magnification (×200) fields were selected to measure the positive staining rates of the cells by different antibodies using Image-Pro Plus 6.0 software (Media Cybernetics, MD, United States).

### Statistical Analysis

We used SPSS 13.0 software (IBM Corporation, Chicago, IL, United States) to calculate statistical results. The data distribution type was confirmed using Kolmogorov–Smirnov test. The numeric result with normal distribution was shown as the mean value with standard deviation (SD). The difference within and between groups were compared by one-way analysis of variance (ANOVA) with Student–Newman–Keuls q test as a *post hoc* test. The correlation strengths between imaging and pathological results were calculated using Pearson correlation analysis. *P*-value < 0.05 was regarded a statistical difference. The Pearson coefficient larger than 0.8 was considered highly correlated, while the coefficient between 0.5 and 0.8 was considered moderately correlated. GraphPad Prism 5.01 (GraphPad Software Inc., San Diego, CA) was used to plot the line charts and scatter charts.

## Results

### Characterization of Nano-Drug and MMT Assay

**FIGURE 1:**
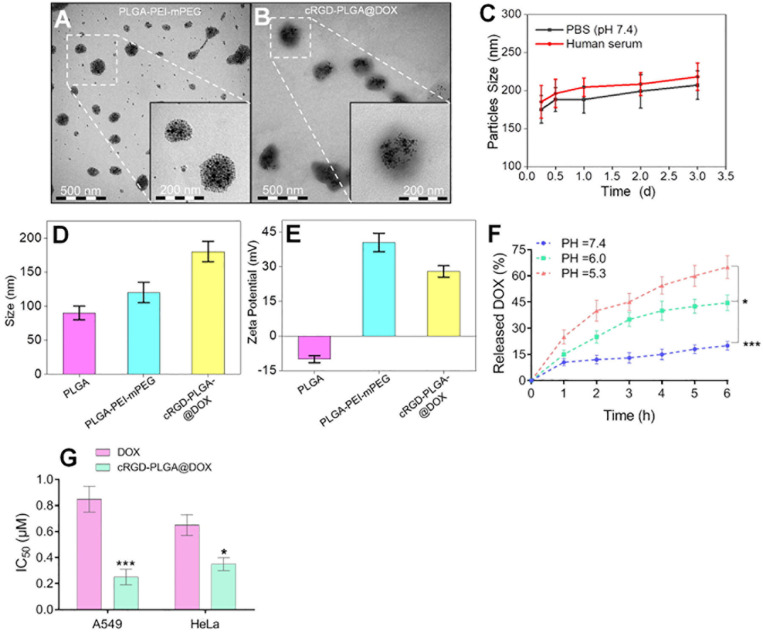
Characterization of cRGD-PLGA@DOX nanoparticles. Transmission electron micrographs of PLGA-PEI-mPEG nanoparticles **(A)** and cRGD-PLGA@DOX nanoparticles **(B)**. The stability examination of the nanoparticle system in PBS and human serum with 3 days **(C)**. The average sizes of PLGA, PLGA-PEI-mPEG, and cRGD-PLGA@DOX copolymers, respectively **(D)**. The zeta potentials of PLGA, PLGA-PEI-mPEG, PLGA-cRGD, and cRGD-PLGA@DOX, respectively **(E)**. The DOX-release behavior of cRGD-PLGA@DOX at pH 7.4, 6.0, and 5.3 in different time points **(F)**. IC50 values of A549 and HeLa cancer cells at a DOX concentration ranging from 0.125 to 8 μM **(G)**. *P < 0.05 and ***P < 0.001.

The composition included 5.0% of cRGD, 60% of polymers, and 33.3% of DOX per nanoparticle. TEM images showed the aminated PLGA-PEI-mPEG and cRGD-PLGA@DOX was homogeneous, well-dispersed, and spherical in shape, with an average size of 120 and 180 nm, respectively (Figures 1A,B,D). Then, the zeta potentials of the PLGA and its derivatives were measured. PEI modification reversed the surface potential of PLGA from −10.0 mV to +40.5 mV. After mPEG and cRGD modification, the positive surface charge of PLGA-PEI was slightly neutralized. After entrapping doxorubicin, the zeta potential slightly decreased to 28.0 mV (Figure 1E). The stability examination of the nanoparticles was shown in Figure 1C, the nanoparticle system remain stable in PBS solution and human serum within 3 days. Furthermore, the DOX-release behavior of cRGD-PLGA@DOX was compared at pH 7.4, 6.0, and 5.3 to mimic the blood circulation environment, tumor microenvironment and acidic environment of lysosomes, respectively. Drug release occurred gradually in a time-dependent pattern without any burst effect (Figure 1F). As shown in Figure 1F, approximately 40 and 60% doxorubicin were released in total after 3 h under conditions mimicking the tumor microenvironment (pH 6.0) and acidic environment of lysosomes (pH 5.3), respectively. By contrast, less than 20% of doxorubicin was released at pH 7.4, indicating a pH-dependent mechanism of release of cRGD-PLGA@DOX. Due to its ability to release doxorubicin⋅HCl in cancer cells while limiting its release in blood circulation, the pH-responsive released behavior of cRGD-PLGA@DOX indicated great potential in drug delivery for anti-proliferative effects. The cytotoxicity of free DOX and cRGD-PLGA@DOX in A549 and HeLa cancer cells were assessed by MTT assays. The DOX concentration of nanosystem needed to kill 50% of the cells (IC50) was 0.25 ± 0.06 vs 0.85 ± 0.10 μM (free DOX group) for A549 cells, and 0.35 ± 0.05 vs 0.65 ± 9.3 μM (free DOX group) for HeLa cells. Taken together, these results indicate that cRGD-PLGA@DOX nanoparticles satisfied the conditions for sustainably and effectively delivering drugs into tumor tissues, which would reduce its toxic side effects against the normal tissues.

### Treatments on Tumor Growth

**FIGURE 2:**
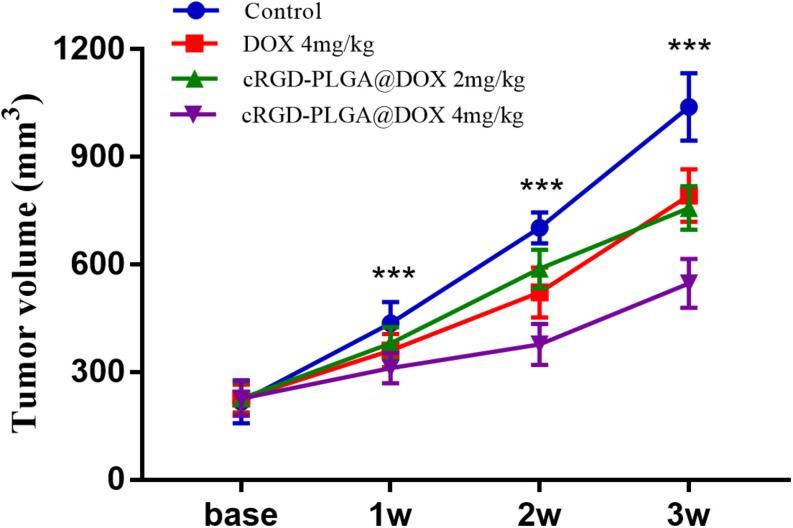
The tumor growth within 3 weeks in Group A, B, C and D. Eight rats were measured and averaged in each group. Group D demonstrated the smallest tumor volume and the highest tumor inhibition rate in 3 weeks, followed by Group C, B, and A. ***P < 0.001.

No mice were died or showed obvious abnormal reaction during the treatment. The results suggested an obvious inhibitory effect on tumor growth in the mice treated with 4 mg/kg of cRGD-PLGA@DOX as Group D demonstrated the smallest tumor volume and the highest tumor inhibition rate in 3 weeks, followed by Group C and B, compared to the control group (Table 1 and Figure 2). The overall tumor inhibition rates in 3 weeks were 23.8, 27.1, and 47.4% in Groups B, C, and D, respectively.

**TABLE 1:** The tumor volume at different time points from the four groups.

Volume (mm^3^)	Base	1 week	2 weeks	3 weeks	*F*	*P*
Group A	217.5 ± 60.1	436.3 ± 59.6	702.5 ± 42.9	1039.8 ± 93.9	225.693	0.001
Group B	226.4 ± 38.9	360.6 ± 45.6	522.0 ± 69.2	792.4 ± 72.8	138.571	0.001
Group C	226.0 ± 45.3	380.5 ± 45.1	588.1 ± 53.4	757.6 ± 60.6	163.686	0.001
Group D	226.9 ± 48.3	311.1 ± 41.9	377.5 ± 56.9	547.4 ± 68.0	49.422	0.001

*Repeated measures analysis of variance was used generate the *P*-value.*

### MRI Results

**FIGURE 3:**
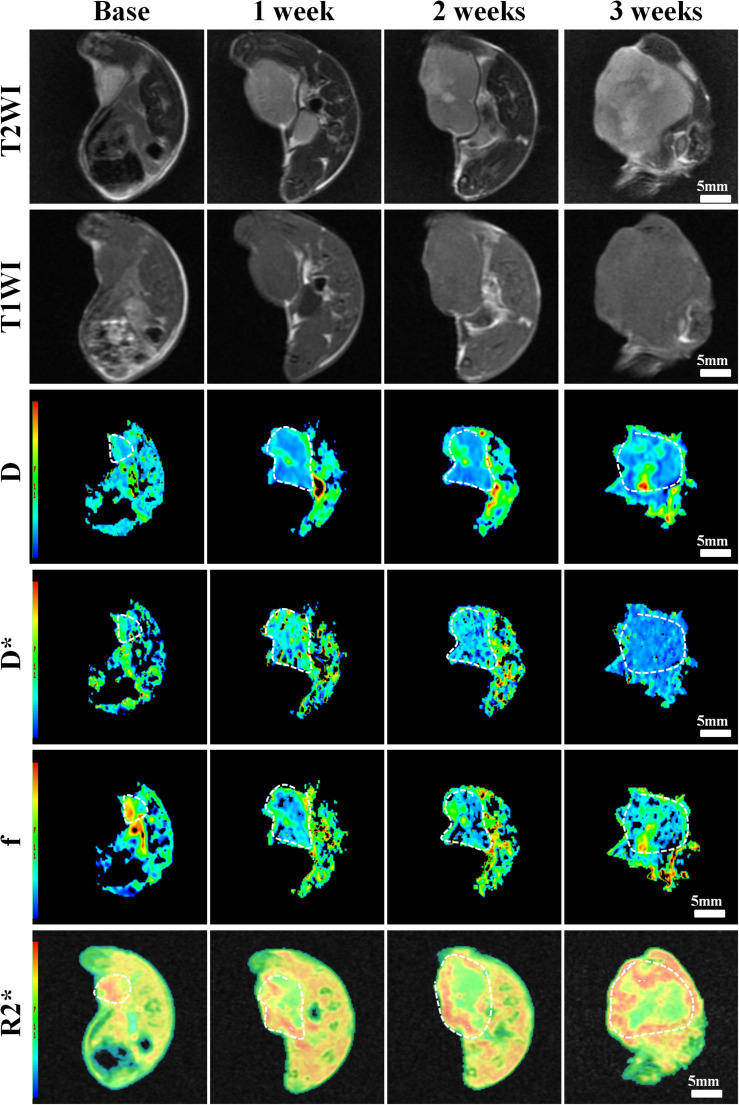
Axial T1WI, T2WI, and pseudocolor maps of D, D*, f, and R2 values at different time points for the control group. D value is the pure diffusion of water molecule. D* value refers to perfusion-related diffusion. F value is the perfusion fraction. R2* value reflects the tumor hypoxia. The white circles plot the tumor area.

**FIGURE 4:**
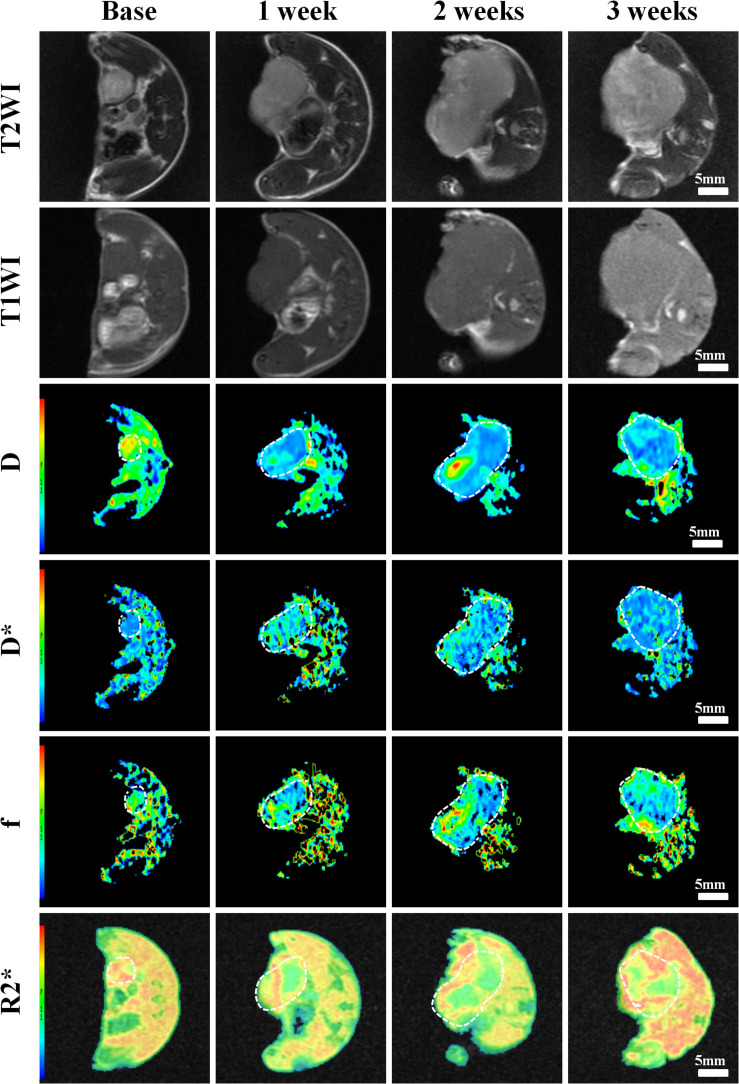
Axial T1WI, T2WI, and pseudocolor maps of D, D*, f, and R2 values at different time points treated with 4 mg/kg of doxorubicin (Group B). D value is the pure diffusion of water molecule. D* value refers to perfusion-related diffusion. F value is the perfusion fraction. R2* value reflects the tumor hypoxia. The white circles plot the tumor area.

**FIGURE 5:**
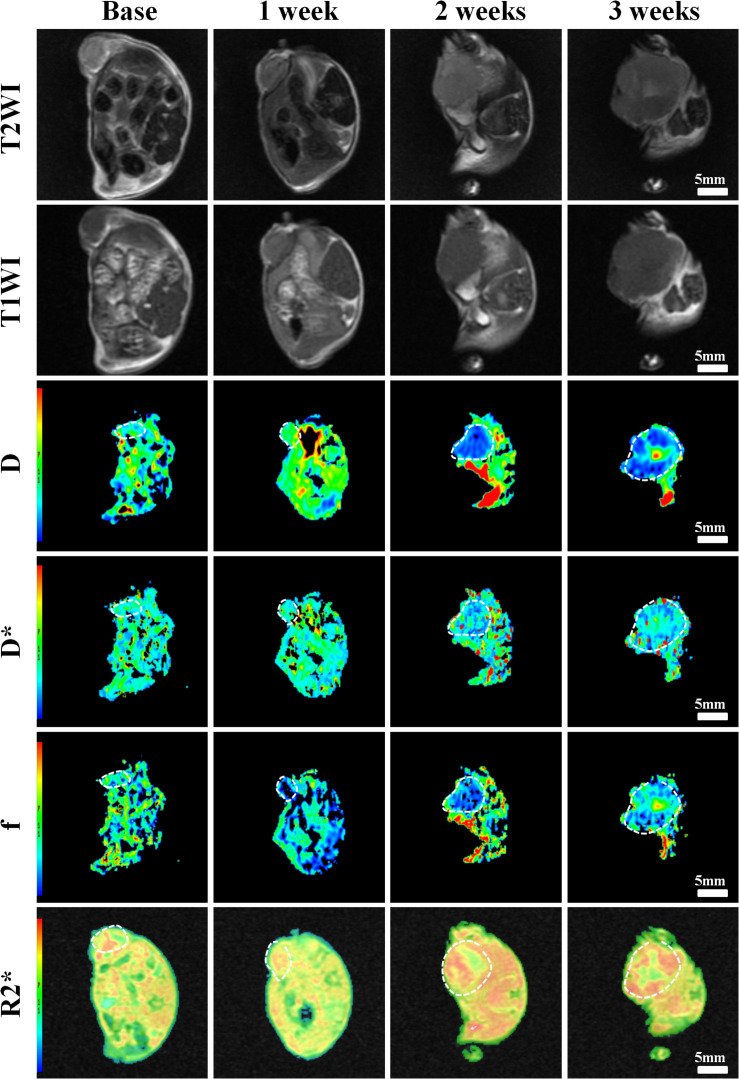
Axial T1WI, T2WI, and pseudocolur maps of D, D*, f, and R2 values at different time points treated with 2 mg/kg of cRGD-PLGA@DOX (Group C). D value is the pure diffusion of water molecule. D* value refers to perfusion-related diffusion. F value is the perfusion fraction. R2* value reflects the tumor hypoxia. The white circles plot the tumor area.

**FIGURE 6:**
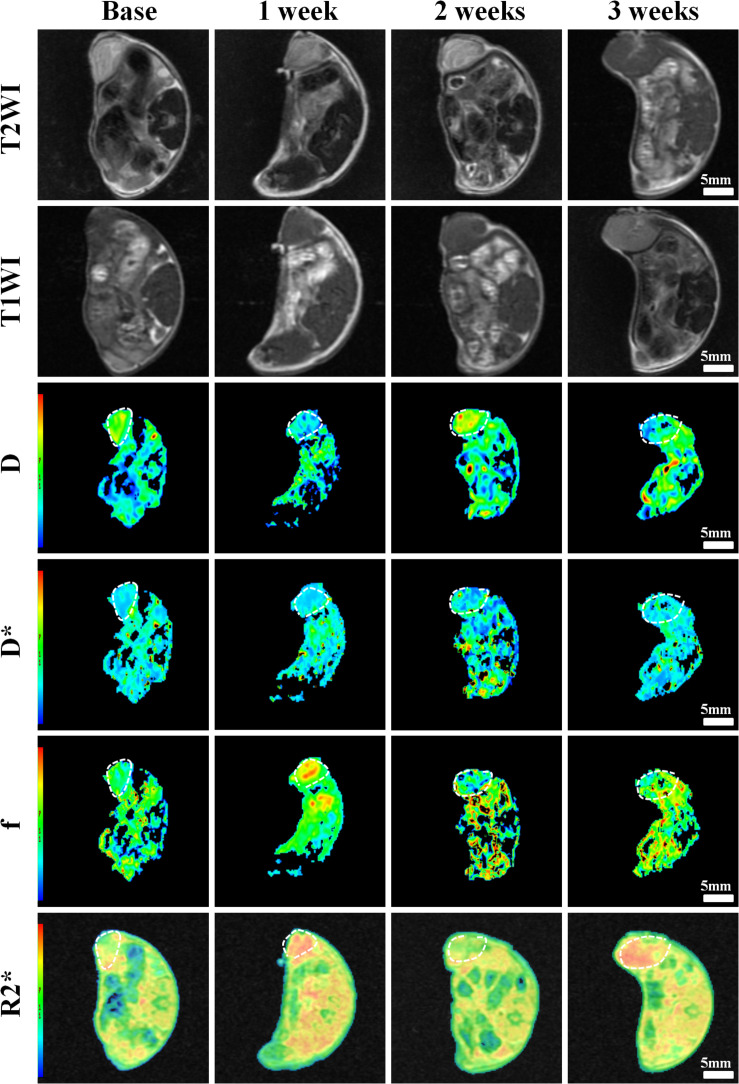
Axial T1WI, T2WI, and pseudocolor maps of D, D*, f, and R2 values at different time points treated with 4 mg/kg of cRGD-PLGA@DOX (Group D). D value is the pure diffusion of water molecule. D* value refers to perfusion-related diffusion. F value is the perfusion fraction. R2* value reflects the tumor hypoxia. The white circles plot the tumor area.

**FIGURE 7:**
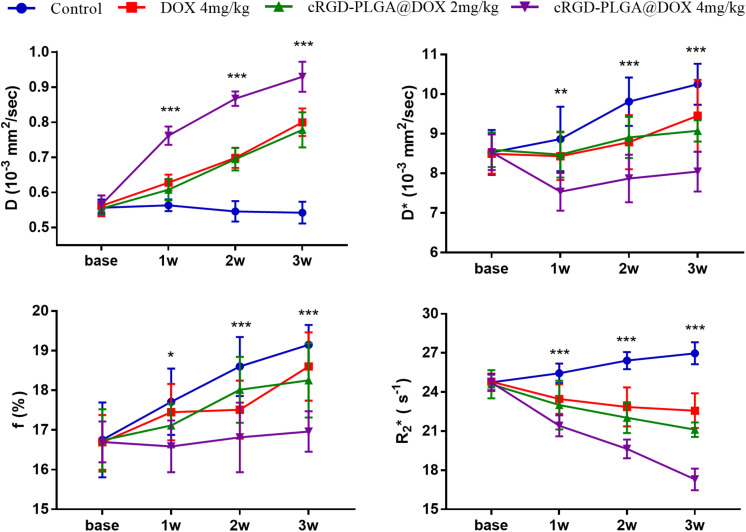
Longitudinal monitoring of IVIM-DWI and R2* mapping in the four groups. The data point indicated mean and standard deviation. *P < 0.05, **P < 0.01, and ***P < 0.001 were generated from comparisons between the four groups at each time point using one-way analysis of variance.

Conventional T1WI, T2WI, and pseudo-color maps of D, D^∗^, f, and R2^∗^ values in Groups A, B, C, D were manifested in Figures 3–6. Their mean values and line charts were manifested in Table 2 and Figure 7. On conventional images, the tumor size in group D was almost unchanged within 3 weeks but with increased patchy of high-signal on T2WI, indicating liquefactive necrosis due to treatment. In contrast, the tumor size of Group A was significantly increased in 3 weeks and already twice larger than that in 1 week, with less high-signal observed on T1WI or T2WI. Obvious high signals were also observed on T2WI in Group B and C, especially at the last two time points, suggesting a moderate efficacy.

**TABLE 2:** The quantitative MRI parameters at different time points from the four groups.

Index	Groups	Base	1 week	2 weeks	3 weeks	*F*	*P*
D (10^–3^mm^2^/s)	Group A	0.556 ± 0.024	0.564 ± 0.017	0.546 ± 0.029	0.536 ± 0.022	2.112	0.121
	Group B	0.562 ± 0.030	0.628 ± 0.023	0.699 ± 0.028	0.800 ± 0.039	88.564	0.001
	Group C	0.555 ± 0.019	0.608 ± 0.031	0.695 ± 0.032	0.778 ± 0.050	64.248	0.001
	Group D	0.566 ± 0.026	0.762 ± 0.026	0.868 ± 0.021	0.930 ± 0.043	227.398	0.001
D* (10^–3^mm^2^/s)	Group A	8.53 ± 0.57	8.87 ± 0.82	9.81 ± 0.61	10.25 ± 0.52	12.594	0.001
	Group B	8.49 ± 0.50	8.43 ± 0.60	8.79 ± 0.68	9.45 ± 0.91	3.643	0.025
	Group C	8.59 ± 0.44	8.47 ± 0.58	8.91 ± 0.52	9.07 ± 0.27	2.792	0.059
	Group D	8.53 ± 0.45	7.54 ± 0.48	7.87 ± 0.60	8.04 ± 0.50	5.307	0.005
f (%)	Group A	16.75 ± 0.95	17.71 ± 0.83	18.60 ± 0.74	19.15 ± 0.50	14.785	0.001
	Group B	16.69 ± 0.69	17.45 ± 0.71	17.51 ± 0.73	18.60 ± 0.86	8.778	0.001
	Group C	16.74 ± 0.79	17.11 ± 0.55	18.01 ± 0.83	18.25 ± 0.94	6.66	0.002
	Group D	16.70 ± 0.51	16.59 ± 0.65	16.81 ± 0.88	16.96 ± 0.51	0.479	0.699
R2* (/s)	Group A	24.74 ± 0.61	25.44 ± 0.75	26.41 ± 0.65	26.98 ± 0.85	15.297	0.001
	Group B	24.79 ± 0.64	23.46 ± 1.14	22.86 ± 1.50	22.58 ± 1.33	5.421	0.005
	Group C	24.60 ± 1.08	23.00 ± 1.87	22.04 ± 1.18	21.11 ± 0.55	11.17	0.001
	Group D	24.74 ± 0.65	21.41 ± 0.81	19.64 ± 0.72	17.30 ± 0.84	136.315	0.001

*Repeated measures analysis of variance was used generate the *P*-value.*

For D value, Group A manifested a slowly decreased trend within 3 weeks, but the results between different time points were insignificant (*F* = 2.112, *P* = 0.121). Group B and C exhibited a similar moderate upward trend within 3 weeks (*P* < 0.001), indicating that the restricted diffusion of water molecules were mildly relieved due to treatments. D value rapidly increased in Group D due to an increase of water diffusion arising from tumor necrosis (*P* < 0.001).

For D^∗^ value, Group A showed a moderate upward trend within 3 weeks (*P* < 0.001), which may result from angiogenesis during the rapid growth period. Both Group B and C showed a slow increased trend within 3 weeks, but a lower D^∗^ value was observed in Group C at the last time point. D^∗^ value obviously decreased in the initial week, but it gradually recovered afterward, indicating an initial tumor necrosis and vascular collapse, followed by tumor regrowth and revascularization. F value, the fraction of pseudo-diffusion, demonstrated similar trends with D^∗^ values among the four groups within 3 weeks with the lowest perfusion level in Group D after treatments, further confirming the superior therapeutic efficacy of this nano-drug compared to the unencapsulated one.

For R2^∗^ value, Group A demonstrated a significant upward trend within 3 weeks (*P* < 0.001), which suggested the tumor was experiencing a hypoxia microenvironment due to the rapid growth period. However, three treatment groups manifested decreased trends with different speeds within 3 weeks (*P* = 0.005, *P* < 0.001, and *P* < 0.001 for Group B, C, and D), and Group D was the most significant with the lowest value.

### Histological Results

**FIGURE 8:**
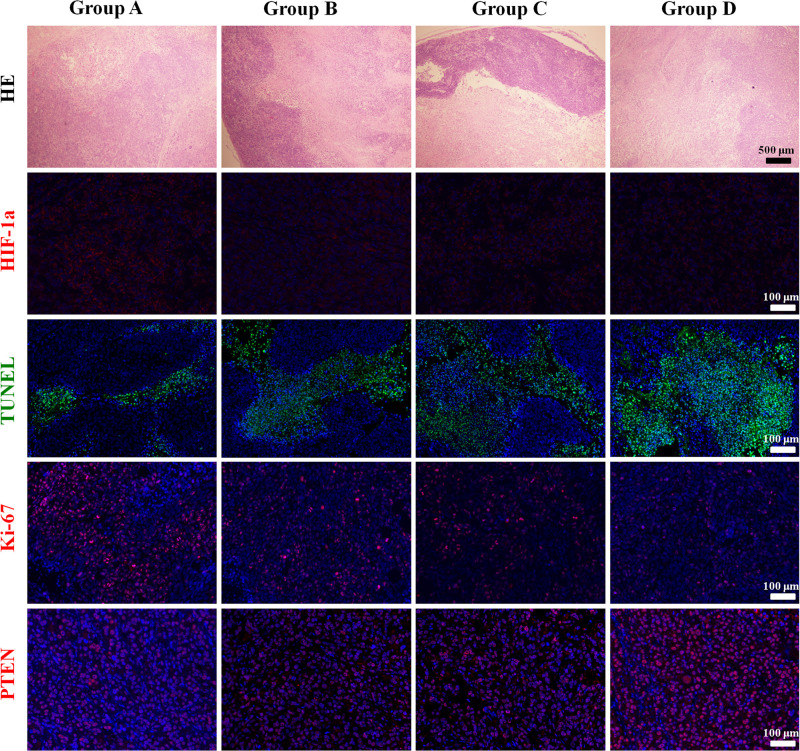
The representative HE, HIF-1α, Ki-67, TUNEL, and PTEN staining in Group A, B, C, and D at the last time point.

**FIGURE 9:**
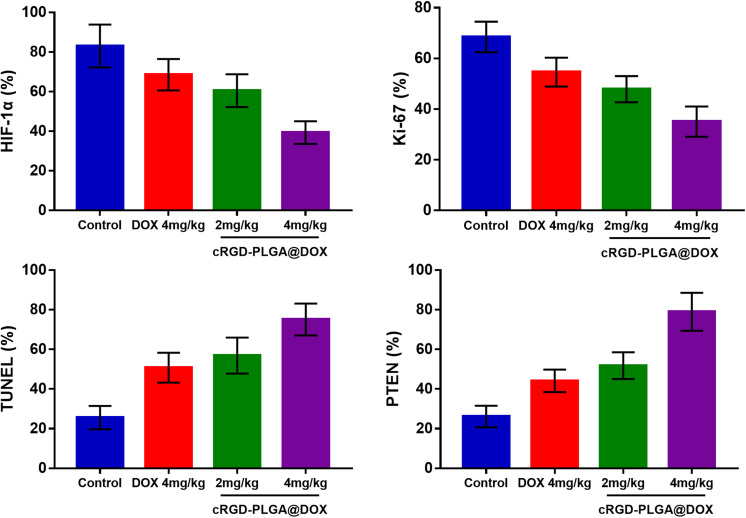
The column charts of pathological indicators in the four groups.

The mean values of pathological indictors in Groups A, B, C, and D at each time point are shown in the Table 3. The representative HE, HIF-1α, Ki-67, TUNEL, and PTEN staining in Group A, B, C, and D at the last time point was shown in Figure 8. Their column charts were manifested in Figure 9. For HE staining, the tumor cells were dense with few of necrosis areas in Group A. In group B and C, a moderate necrotic areas and patches of hemorrhage occurred in the tumor cores. More karyopyknosis and nuclear fragmentation with homogeneous red-stained areas were observed in Group D. All the treatment groups demonstrated a reduced HIF-1α expression compared to the control group, with the lowest value in Group D (*F* = 37.969, *P* < 0.001), which may result from the relief of tumor hypoxia due to necrosis. Ki-67 staining also suggested a decreased Ki-67 expression in the treatment groups due to inhibition by doxorubicin, and the nanoparticle may enhance the drug delivery and inhibited effect (*F* = 47.414, *P* < 0.001). TUNEL staining demonstrated a moderate area of apoptotic cells in Group B and C and a larger area of apoptosis in Group D, compared to the control group (*F* = 55.906, *P* < 0.001). Group D showed the highest PTEN expression among the four groups, followed by Group C, B, and A (*F* = 77.738, *P* < 0.001), suggesting a recovered expression of tumor suppressor gene that inhibits tumor growth.

**TABLE 3:** The pathological indictors at the last time point from the four groups.

Indictor	Group A	Group B	Group C	Group D	*F*	*P*
HIF-1a (%)	83.1 ± 10.8	68.6 ± 7.9	60.5 ± 8.3	39.3 ± 5.7	37.969	0.001
Ki-67 (%)	68.5 ± 6.0	54.6 ± 5.7	47.9 ± 5.1	35.1 ± 5.9	47.414	0.001
TUNEL (%)	25.6 ± 5.9	50.8 ± 7.6	56.9 ± 9.1	75.1 ± 8.1	55.906	0.001
PTEN (%)	26.1 ± 5.4	44.1 ± 5.7	51.8 ± 6.7	79.0 ± 9.6	77.738	0.001

*One-way analysis of variance was used generate the *P*-value. HIF-1α, hypoxia-inducible factor 1α; TUNEL, terminal-deoxynucleoitidyl transferase mediated nick end labeling; PTEN, phosphate and tension homology deleted on chromosome ten.*

### Correlation Results

**FIGURE 10:**
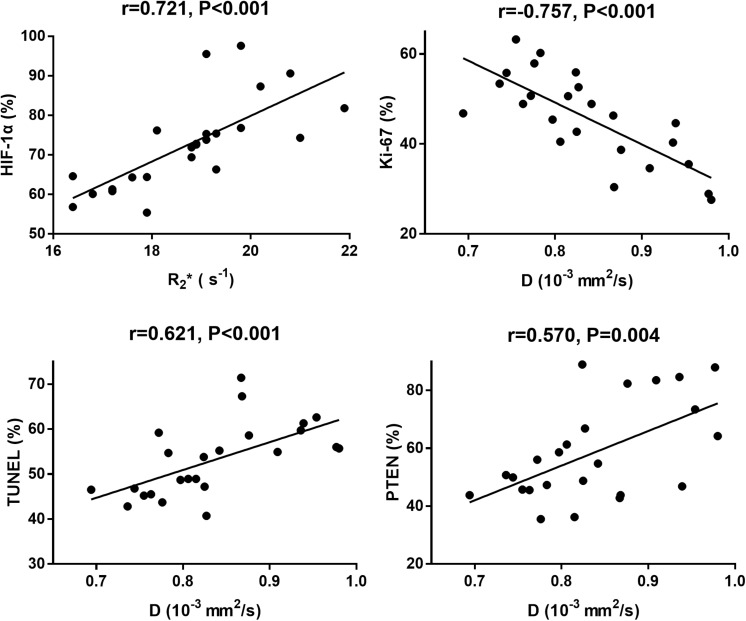
The scatter chars plotted the highest correlations between MRI parameters and each pathological indicator.

The correlation coefficients between imaging and pathological indicators were listed in Table 4. The scatter charts were plotted in Figure 10. In reflecting tumor hypoxia, HIF-1α was positively correlated with R2^∗^ value (*r* = 0.721, *P* < 0.001) but inversely correlated with D^∗^ (*r* = −0.582, *P* = 0.003) and f values (*r* = −0.609, *P* = 0.002). In assessing cell proliferation activity, Ki-67 was positively correlated with R2^∗^ value (*r* = 0.416, *P* = 0.043) but inversely correlated with D value (*r* = −0.757, *P* < 0.001). In assessing cell apoptosis, TUNEL staining was only positively correlated with D value (*r* = 0.621, *P* < 0.001). PTEN staining was positively correlated with both D (*r* = 0.57, *P* = 0.004) and f values (*r* = 0.428, *P* = 0.037), but their associated strengths were weak to moderate.

**TABLE 4:** Pearson correlation coefficients between imaging and pathological parameters.

Pearson coefficient	D		D*		f		R2*	
	*r*	*P*	*r*	*P*	*r*	*P*	*r*	*P*
HIF-1a	0.322	0.125	−0.582	0.003	−0.609	0.002	0.721	0.001
Ki-67	−0.757	0.001	−0.348	0.096	−0.209	0.328	0.416	0.043
TUNEL	0.621	0.001	0.208	0.329	0.248	0.243	0.228	0.285
PTEN	0.57	0.004	0.405	0.05	0.428	0.037	0.39	0.059

*HIF-1α, hypoxia-inducible factor 1α; TUNEL, terminal-deoxynucleoitidyl transferase mediated nick end labeling; PTEN, phosphate and tension homology deleted on chromosome ten. The values in bold indicate statistically significant.*

## Discussion

Monitoring the tumor microenvironment is an important direction of current anti-tumor therapy, including the degree of tumor hypoxia, cellularity and microcirculation perfusion, which have close relationships with each other and determine the efficacy of nanomedicine. In this study, we functionalized the nanocarrier by coating the surface with PEG. On the one hand, the polymer PEG as a hydrophilic surface coating at the nanocarrier, can increase the carrier’s escape ability from macrophages and reticuloendothelial system, and improve the circulation time of the carriers in the body. On the other hand, a functional modification with cRGD on the surface can further increase the active recognition ability of the nanocarriers. In other to confirm the therapeutic efficacy of this nano-drug, we monitored the microenvironmental changes using two non-invasive MR sequences and compared with several pathological staining. The results provided a more reliable basis for MRI to further evaluate the efficacy of nanomedicine *in vivo* in cancer treatment.

In this study, we found that Group D showed the smallest tumor volume with the tumor inhibition rate of 47.4%, suggesting a significant inhibition on the tumor growth when treated with 4 mg/kg of cRGD-PLGA@DOX, which may result from the selective delivery induced by RGD-mediated active targeting property, and enhanced permeability and retention effects of the tumor vessels. Whereas the mice treated with the same dose of doxorubicin only manifested an inhibition rate of 23.8%, even lower than the ones treated with 2 mg/kg of cRGD-PLGA@DOX (27.1%), suggesting the low effectiveness of unencapsulated doxorubicin. The higher Ki-67 expression had been reported to be related with a high risk of metastasis and recurrence, poor clinical and pathological response, and worse survival (21). It has been widely used in clinical practice as an ideal predictive and prognostic marker for cancer treatment. Therefore, a decreased Ki-67 expression in the treatment groups indicated an improved therapeutic outcome. Besides, a larger area of necrosis indicated by HE staining and conventional MRI, an increased percentage of apoptotic cells and a recovery of PTEN expression across the tumor sections further confirmed that nanomedicine can significantly improve the therapeutic effect or reduce the tissue toxicity using a lower dosage.

Only evaluating the tumor volumes was not enough to fully reflect the treatment efficacy of the functional particle as changes in tumor volume often lag behind functional changes such as tumor perfusion, hypoxia, and cell activity. In this study, we used IVIM-DWI and R2^∗^ mapping to dynamically monitor the microenvironment changes and found that D value significantly increased over time in Group D, followed by Groups B and C. D value reflects the diffusion rate of water molecule, which may be limited in the crowded tumor cellularity with high proliferative activity but relieved in the necrotic tumor due to treatments. Increased Ki-67 expression suggested higher cellularity and active vascular hyperplasia. In this study, D value exhibited negative and moderate correlation with Ki-67 (*r* = −0.757), positive correlations with TUNEL (*r* = 0.621) and PTEN (*r* = 0.570), indicating D value can be used to predict the treatment efficacy.

Furthermore, both D^∗^ and f values shared a similar increased trend but with an inhibited speed in Group B and C, compared with control group. D^∗^ and f values are perfusion-related parameters that reflect the microcirculation perfusion within the tumor microvasculature (8). Although doxorubicin cannot directly destroy the existing tumor microvasculature and block the blood supply, it can inhibit the expression of several transcription factors in addition to killing the tumor cells with high tissue penetrability (22, 23). The expressions of vascular endothelial growth factors and other pro-angiogenic factors were subsequently down-regulated, reducing the microvessel density by indirect inhibition of the tumor angiogenesis (24, 25). The perfusion parameters even decreased in the initial week but slightly recovered afterward in Group D, suggesting the nanoparticle performed a much strong inhibited effect on the tumor angiogenesis in a short time, but the perfusion was slightly recovered thereafter due to the revascularization in the tumor edge.

R2^∗^ mapping is an advanced sequence that sensitive to deoxyhemoglobin, and commonly used to reflect the tumor hypoxia (26). An increased R2^∗^ value signifies the presence of hypoxia due to tumor growth. In our study, all the treatment groups demonstrated a decreased R2^∗^ value compared to the control group. Although the tumor angiogenesis was inhibited by doxorubicin and the blood supply decreased to a certain extent, the subsequent tumor necrosis and apoptosis will reduce the demand for oxygen supply and relieve the degree of hypoxia. Therefore a lower R2^∗^ value indicated more effective anti-cancer treatment in Group D. What’s more, R2^∗^ value revealed the highest correlation with HIF-1α (*r* = 0.721), a pathological indicator for evaluating tumor hypoxia.

There are some limitations in this study. First, the pathological changes at other time points were unknown due to lack of enough tumor samples. Second, we only delineated the regions of interest at the largest tumor sections for calculation while the whole tumor lesions should be considered to ensure the stability and reliability of the measurement. Last, we have not performed dynamic contrast-enhanced MRI which is the gold standard method for perfusion imaging, and compared the diagnostic performance with IVIM-DWI. The combination imaging should be considered in the future study.

## Conclusion

We encapsulated the doxorubicin with the polymer PEG-PLGA which was conjugated with a targeted ligand cRGD. The nanocarriers demonstrated a higher utilization efficiency and therapeutic efficacy for doxorubicin in treating lung cancer due to the active targeting property and enhanced permeability and retention effect. IVIM-DWI and R2^∗^ mapping are two valuable sequences that can dynamically monitor the tumor hypoxia, cellularity, and microcirculation perfusion during treatment; the returned information may help to further understand the anti-cancer mechanisms of nanomedicine and adjust the treatment in time.

## Data Availability Statement

The raw data supporting the conclusions of this article will be made available by the authors, without undue reservation.

## Ethics Statement

The animal study was reviewed and approved by the Institutional Animal Ethics Committee of Jinan University.

## Author Contributions

NS, ZX, and LL: conceptualization, writing – review, editing, and approving. CH, JL, and MM: animal experiment and writing – original draft preparation. QC and XX: MRI examinations. DZ and CS: pathological analysis. All authors contributed to the article and approved the submitted version.

## Conflict of Interest

The authors declare that the research was conducted in the absence of any commercial or financial relationships that could be construed as a potential conflict of interest.

## Abbreviations

D: true diffusion coefficient
D^∗^: pseudo-diffusion coefficient
DOX: doxorubicin
f: perfusion fraction
HIF-1 α: hypoxia-inducible factor 1 α
IVIM-DWI: intravoxel incoherent motion diffusion-weighed imaging
MRI: magnetic resonance imaging
PEG: polyethylene glycol
PLGA: poly-(lactic-co-glycolic acid)
RGD: arginine-glycine-aspartic acid polypeptide
R2^∗^: transverse relaxation rate.

## References

[1] Bray F Ferlay J Soerjomataram I Siegel RL Torre LA Jemal A. Global cancer statistics 2018: GLOBOCAN estimates of incidence and mortality worldwide for 36 cancers in 185 countries. *CA Cancer J Clin.* (2018) 68:394–424. 10.3322/caac.21492 30207593

[2] Gao P Mei C He L Xiao Z Chan L Zhang D Designing multifunctional cancer-targeted nanosystem for magnetic resonance molecular imaging-guided theranostics of lung cancer. *Drug Deliv.* (2018) 25:1811–25. 10.1080/10717544.2018.1494224 30465437 PMC6263109

[3] Xiao Z Chan L Zhang D Huang C Mei C Gao P Precise delivery of a multifunctional nanosystem for MRI-guided cancer therapy and monitoring of tumor response by functional diffusion-weighted MRI. *J Mater Chem B.* (2019) 7:2926–37. 10.1039/c8tb03153c

[4] Tanner P Baumann P Enea R Onaca O Palivan C Meier W. Polymeric vesicles: from drug carriers to nanoreactors and artificial organelles. *Acc Chem Res.* (2011) 44:1039–49. 10.1021/ar200036k 21608994

[5] Yoo HS Lee EA Park TG. Doxorubicin-conjugated biodegradable polymeric micelles having acid-cleavable linkages. *J Control Release.* (2002) 82:17–27. 10.1016/s0168-3659(02)00088-3 12106973

[6] Le Bihan D Breton E Lallemand D Grenier P Cabanis E Laval-Jeantet M. MR imaging of intravoxel incoherent motions: application to diffusion and perfusion in neurologic disorders. *Radiology.* (1986) 161:401–7. 10.1148/radiology.161.2.3763909 3763909

[7] Pan JH Zhu S Huang J Liang J Zhang D Zhao X Monitoring the process of endostar-induced tumor vascular normalization by non-contrast intravoxel incoherent motion diffusion-weighted MRI. *Front Oncol.* (2018) 8:524. 10.3389/fonc.2018.00524 30483478 PMC6243029

[8] Gao P Liu Y Shi C Liu Y Luo L. Performing IVIM-DWI using the multifunctional nanosystem for the evaluation of the antitumor microcirculation changes. *MAGMA.* (2020) 33:517–26. 10.1007/s10334-019-00814-7 31897903

[9] Liang J Song X Xiao Z Chen H Shi C Luo L. Using IVIM-MRI and R2 mapping to differentiate early stage liver fibrosis in a rat model of radiation-induced liver fibrosis. *Biomed Res Int.* (2018) 2018:4673814. 10.1155/2018/4673814 30627558 PMC6304485

[10] Liang J Ma R Chen H Zhang D Ye W Shi C Detection of hyperacute reactions of desacetylvinblastine monohydrazide in a xenograft model using intravoxel incoherent motion DWI and R2^∗^ mapping. *AJR Am J Roentgenol.* (2019) 212:717–26. 10.2214/AJR.18.20517 30699010

[11] Shi C Liu D Xiao Z Zhang D Liu G Liu G Monitoring tumor response to antivascular therapy using non-contrast intravoxel incoherent motion diffusion-weighted MRI. *Cancer Res.* (2017) 77:3491–501. 10.1158/0008-5472.CAN-16-2499 28487383

[12] Liang J Cheng Q Huang J Ma M Zhang D Lei X Monitoring tumour microenvironment changes during anti-angiogenesis therapy using functional MRI. *Angiogenesis.* (2019) 22:457–70. 10.1007/s10456-019-09670-4 31147887

[13] Yang N Ding Y Zhang Y Wang B Zhao X Cheng K Surface functionalization of polymeric nanoparticles with umbilical cord-derived mesenchymal stem cell membrane for tumor-targeted therapy. *ACS Appl Mater Interfaces.* (2018) 10:22963–73. 10.1021/acsami.8b05363 29905067

[14] Palui G Aldeek F Wang W Mattoussi H. Strategies for interfacing inorganic nanocrystals with biological systems based on polymer-coating. *Chem Soc Rev.* (2015) 44:193–227. 10.1039/c4cs00124a 25029116

[15] Ermolayev V Mohajerani P Ale A Sarantopoulos A Aichler M Kayser G Early recognition of lung cancer by integrin targeted imaging in K-ras mouse model. *Int J Cancer.* (2015) 137:1107–18. 10.1002/ijc.29372 25450481

[16] Desgrosellier JS Cheresh DA. Integrins in cancer: biological implications and therapeutic opportunities. *Nat Rev Cancer.* (2010) 10:9–22. 10.1038/nrc2748 20029421 PMC4383089

[17] Lei X Chen M Huang M Li X Shi C Zhang D Desacetylvinblastine monohydrazide disrupts tumor vessels by promoting VE-cadherin internalization. *Theranostics.* (2018) 8:384–98. 10.7150/thno.22222 29290815 PMC5743555

[18] Cho GY Moy L Kim SG Baete SH Moccaldi M Babb JS Evaluation of breast cancer using intravoxel incoherent motion (IVIM) histogram analysis: comparison with malignant status, histological subtype, and molecular prognostic factors. *Eur Radiol.* (2016) 26:2547–58. 10.1007/s00330-015-4087-3 26615557 PMC4894831

[19] Robinson SP Rodrigues LM Howe FA Stubbs M Griffiths JR. Effects of different levels of hypercapnic hyperoxia on tumour R(2)^∗^ and arterial blood gases. *Magn Reson Imaging.* (2001) 19:161–6. 10.1016/s0730-725x(01)00230-2 11358653

[20] Li F Lee KE Simon MC. Detection of hypoxia and HIF in paraffin-embedded tumor tissues. *Methods Mol Biol.* (2018) 1742:277–82. 10.1007/978-1-4939-7665-2_24 29330808 PMC6002766

[21] Jiang S Hong YJ Zhang F Li YK. Computer-aided evaluation of the correlation between MRI morphology and immunohistochemical biomarkers or molecular subtypes in breast cancer. *Sci Rep.* (2017) 7:13818. 10.1038/s41598-017-14274-3 29062076 PMC5653801

[22] Morrison PF Laske DW Bobo H Oldfield EH Dedrick RL. High-flow microinfusion: tissue penetration and pharmacodynamics. *Am J Physiol.* (1994) 266:R292–305. 10.1152/ajpregu.1994.266.1.R292 8304553

[23] Johnson DG Schwarz JK Cress WD Nevins JR. Expression of transcription factor E2F1 induces quiescent cells to enter S phase. *Nature.* (1993) 365:349–52. 10.1038/365349a0 8377827

[24] Friedli A Fischer E Novak-Hofer I Cohrs S Ballmer-Hofer K Schubiger PA The soluble form of the cancer-associated L1 cell adhesion molecule is a pro-angiogenic factor. *Int J Biochem Cell Biol.* (2009) 41:1572–80. 10.1016/j.biocel.2009.01.006 19401151

[25] Fukumura D Jain RK. Tumor microvasculature and microenvironment: targets for anti-angiogenesis and normalization. *Microvasc Res.* (2007) 74:72–84. 10.1016/j.mvr.2007.05.003 17560615 PMC2100036

[26] Wu G Liu G Kong W Qu J Suo S Liu X Assessment of response to anti-angiogenic targeted therapy in pulmonary metastatic renal cell carcinoma: R2^∗^ value as a predictive biomarker. *Eur Radiol.* (2017) 27:3574–82. 10.1007/s00330-016-4700-0 28130612

